# Correlations between endometriosis, lipid profile, and estrogen levels

**DOI:** 10.1097/MD.0000000000034348

**Published:** 2023-07-21

**Authors:** Rong Zheng, Xin Du, Yan Lei

**Affiliations:** a Department of Gynecology, Maternal and Child Health Hospital of Hubei Province, Tongji Medical College, Huazhong University of Science and Technology, Wuhan, Hubei, China.

**Keywords:** blood lipid, EMS, estrogen

## Abstract

To explore the association between serum lipids and the occurrence and development of endometriosis using a retrospective review of clinical data. A total of 177 patients who underwent laparoscopic or open surgery due to benign ovarian masses, 117 patients with endometriosis (53 stage III and 64 stage IV), and 60 patients with benign ovarian masses without endometriosis were selected from the gynecology department of Maternal and Child Health Hospital of Hubei Province, between January 1, 2020, and October 30, 2022, to search for endometriosis occurrence by retrospectively analyzed the patients clinical data Risk factors for development and to explore the relationship between blood lipids and endometriosis. The scores of estradiol (E2), carbohydrate antigen 125 (CA125), and pain in the endo - and non-endometriosis groups were significantly different (*P* < .05), but there was no significant correlation between these 3. There were significant differences (*P* < .05) in E2, triglyceride (TG), CA125, and the size of the masses between patients with stage III and IV endometriosis. TG, E2, and CA125 were found to be valuable as separate indicators for the prediction of endometriosis, and the 3 indicators could improve the accuracy of the diagnosis of endometriosis when combined. Triglycerides may be positively correlated with the severity of endometriosis. The combination of TG, E2 and CA125 can improve the accuracy of the diagnosis of endometriosis staging.

## 1. Introduction

Endometriosis (EMS) is a common, multimorbidity condition in women of reproductive age, which is defined by the presence of endometrial tissue (glandular and stromal) in the uterine cavity overlying the endometrium and elsewhere than the uterus, which subsequently triggers dysmenorrhea, menstrual abnormalities, dyspareunia, infertility, etc, affecting approximately 10% of women and increasing the expenditure of social medical care.^[1]^ EMS is characterized by estrogen dependence, the pathogenesis of which remains undefined. Lipids are not only important components of the structure of living organisms, but also participate in multiple signaling pathways, which affect the function of cells to varying degrees, and abnormal lipid metabolism is involved in processes such as inflammation, immunity, and steroid hormone metabolism, and the relationship with the occurrence and development of some diseases has been of great interest.^[2]^ This study aimed to investigate the relationship between estrogen, lipid profile, body mass index (BMI), and the occurrence and development of EMS through a retrospective review of patients clinical data.

## 2. Methods

### 2.1. Study subjects

A total of 177 patients treated by laparoscopic or open surgery due to benign ovarian masses from January 1, 2020, to October 30, 2022, in the gynecology department of Maternal and Child Health Hospital of Hubei Province were selected. Our data comes from the hospital’s data system, which can obtain and extract data from the hospital’s internal computer system. Based on the surgical findings and the postoperative pathological diagnosis, 117 patients had postoperative pathologically confirmed EMS (53 stage III and 64 stage IV), and 60 patients had benign masses in the adnexal area without EMS. Inclusion criteria: premenopausal women aged 18 to 49 years; Did not receive hormone therapy within 1 month before treatment; Had no previous history of autoimmune disease or malignancy; No hypertension, diabetes mellitus, hyperthyroidism, and other endocrine diseases related to lipid metabolism; Patients with first diagnosed, no previous surgical history of the same disease. Exclusion criteria: postoperative pathological diagnosis of malignancy; Comorbid pregnancy-related disorders; Concurrent cervical cancerous or precancerous lesions; Patients who refused surgical treatment for whom pathological results could not be obtained. This study has been discussed and adopted by the Ethics Committee of Maternal and Child Health Hospital of Hubei Province, and all patients gave written informed consent before treatment. Patients or the public were not involved in the design, conduct, reporting or dissemination plans of our research.

### 2.2. Methods

Height (m), weight (kg), and BMI = weight (kg)/height2 (m2) were measured on admission for all patients, while clinical characteristics including age, number of pregnancies, lipids [triglycerides, cholesterol, high-density lipoprotein (HDL), low-density lipoprotein (LDL)], estradiol (E2), anti-Mullerian hormone, glycoantigen 125 (CA125), blood group Pain scores during dysmenorrhea (the pain rating tool selected was the numerical rating scale,^[3]^ which uses a scale from 0 to 10, with 0 representing no pain and 10 representing the worst pain imaginable), the maximum diameter of the adnexal mass, and the presence or absence of comorbid fibroids, endometrial polyps, adenomyosis diagnosed radiographically or surgically by ASRM staging in patients with EMS. Differences in clinical data between the EMS group, non-EMS group, and patients with stage III or IV EMS were compared, and risk factors for the occurrence and development of EMS were analyzed.

Statistical analyses were performed using SPSS 22.0. Count data were expressed as the number of cases (constituent ratio) and were analyzed differentially using χ 2-test, the measurement data were expressed as x ± s by those who met the normal distribution, F-test was used for the difference analysis, and ANOVA was used for comparison between groups, univariate logistic regression analysis was performed for each clinical indicator, and *P* < .05 was considered statistically significant. No adjusted analysis for potential confound-ing factors was performed when comparing the different group sand sub groups. Scatter plots were used for correlation studies, and receiver operating characteristic (ROC) curves were used for the predictive value of the disease. ROC curves were constructed to evaluate the predictive power of EMS staging based on area under the curve (AUC) and 95% confidence interval.

## 3. Results

### 3.1. Comparison of clinical characteristics between EMS and non-EMS patients

**Figure 1.**
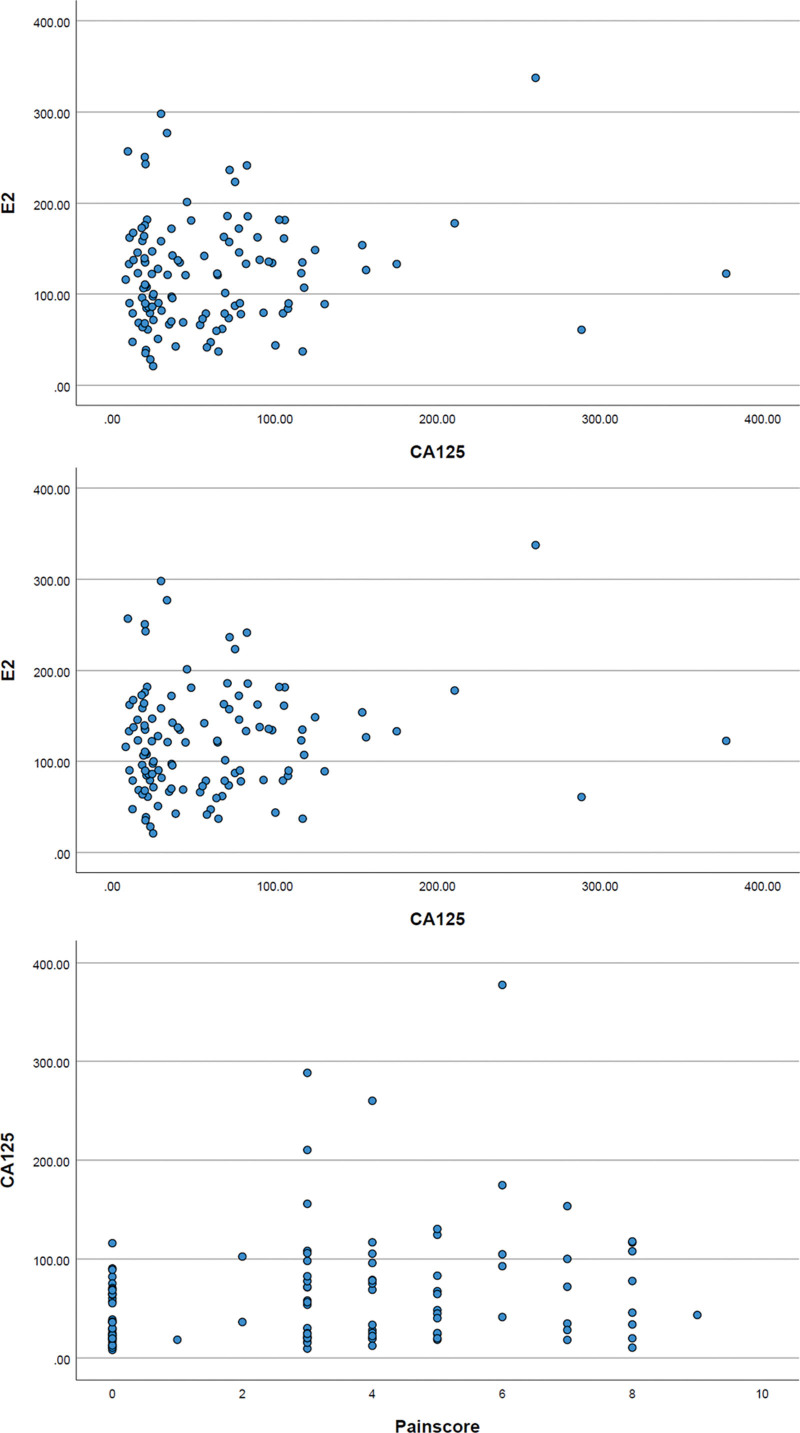
Scatter plots of E2, CA125 and pain scores. Scatter plots showed no significant correlations between E2, CA125 and pain scores. E2 = Estradiol, CA125 = carbohydrate antigen 125.

There were significant differences in the E2, CA125, and pain scores between the EMS and non-EMS groups (*P* < .05), while there were no significant differences in age, lipid profile, number of pregnancies, BMI, blood group, size of the mass, presence or absence of comorbid fibroids, endometrial polyps, adenomyosis (*P* > .05), Table 1. Scatter plots showed no significant correlations between E2, CA125 and pain scores (Fig. 1).

**Table 1:** Comparison of clinical characteristics between EMS and non-EMS patients.

Clinical characteristics	EMS	Non-EMS	X^2^/t	*P* value
Category	n = 117	n = 60		
Age (yr, χ2 ± s)	36.06 ± 7.21	36.22 ± 7.11	−0.14	.891
E2 (pg/mL, χ2 ± s)	121.76 ± 59.41	94.49 ± 56.68	2.98	.003
TC (mmol/L, χ2 ± s)	4.19 ± 0.73	4.16 ± 0.85	0.118	.906
TG (mmol/L, χ2 ± s)	1.12 ± 0.68	1.23 ± 0.88	−0.84	.406
LDL (mmol/L, χ2 ± s)	2.61 ± 0.67	2.67 ± 0.75	−0.49	.626
HDL (mmol/L, χ2 ± s)	1.32 ± 0.32	1.28 ± 0.27	0.95	.344
CA125 (U/mL, χ2 ± s)	60.00 ± 57.32	25.72 ± 29.95	4.33	.000
BMI (kg/m2, χ2 ± s)	23.07 ± 3.53	23.43 ± 3.76	−0.61	.542
AMH (ng/mL, χ2 ± s)	2.20 ± 2.40	2.14 ± 2.10	0.18	.855
Pain scores (χ2 ± s)	3.04 ± 2.62	0.58 ± 1.49	6.72	.000
Size of the mass (cm, χ2 ± s)	6.64 ± 1.94	7.63 ± 5.58	−1.72	.088
Number of pregnancies (times, χ2 ± s)	1.66 ± 1.46	2.00 ± 1.61	−1.38	.169
Blood groups				
A	46	20		
B	24	18	0.99	.922
AB	10	4		
O	37	18		
Presence or absence of associated fibroids [n (%)]				
Yes	76 (64.96%)	47 (78.33%)	3.35	.085
No	41 (35.04%)	13 (21.64%)		
Presence or absence of Endometrial polyps [n (%)]			0.53	.563
No	90 (76.92%)	49 (81.67%)		
Yes	27 (23.08%)	11 (18.33%)		
Presence or absence of adenomyosis [n (%)]				.163
No	90 (76.92%)	52 (86.67%)	2.37	
Yes	27 (23.78%)	8 (13.33%)		

The differences in E2, CA125, pain score, age, blood lipid, number of pregnancies, BMI, blood type, mass size, uterine fibroids, endometrial polyps and adenomyosis were compared between the 2 groups. There were significant differences in the E2, CA125, and pain scores between the EMS and non-EMS groups.

AMH = Anti-Mullerian Hormone, BMI = Body Mass Index, CA125 = Carbohydrate antigen 125, E2 = Estradiol, EMS = Endometriosis, HDL = High-Density Lipoprotein, LDL = Low-Density Lipoprotein, Non-EMS = non-Endometriosis, TC = Total Cholesterol, TG = Triglyceride.

### 3.2. Comparison of clinical characteristics among EMS patients with different stages

The E2, triglyceride (TG), CA125, and size of the tumor mass from stage III and IV EMS patients were significantly different (*P* < .05), whereas age, total cholesterol (TC), LDL, HDL, number of pregnancies, BMI, blood group, presence or absence of associated fibroids, endometrial polyps, and adenomyosis were not statistically different (*P* > .05). Shown in Table 2.

**Table 2:** Comparison of clinical characteristics between stage III and IV EMS patients.

Clinical characteristics	Stage III	Stage IV	X^2^/t	*P* value
Category	n = 53	n = 64		
Age (yr, χ2 ± s)	36.87 ± 7.31	35.39 ± 7.11	1.10	.273
E2 (pg/mL, χ2 ± s)	99.91 ± 51.00	139.86 ± 60.14	−3.89	.000
TC (mmol/L, χ2 ± s)	4.17 ± 0.75	4.21 ± 0.72	−0.27	.788
TG (mmol/L, χ2 ± s)	0.96 ± 046	1.26 ± 0.80	−2.41	.018
LDL (mmol/L, χ2 ± s)	2.62 ± 0.73	2.61 ± 0.63	0.138	.890
HDL (mmol/L, χ2 ± s)	1.34 ± 0.29	1.30 ± 0.34	0.67	.501
CA125 (U/mL, χ2 ± s)	44.52 ± 55.56	72.80 ± 55.99	−2.73	.07
BMI (kg/m2, χ2 ± s)	23.26 ± 3.76	22.92 ± 3.34	0.51	.613
AMH (ng/mL, χ2 ± s)	2.02 ± 2.32	2.35 ± 2.47	−0.75	.455
Pain scores (χ2 ± s)	2.77 ± 2.55	3.27 ± 2.69	−1.02	.312
Size of the mass (cm, χ2 ± s)	6.16 ± 1.71	7.05 ± 2.03	−2.58	.011
Number of pregnancies (times, χ2 ± s)	1.94 ± 1.42	1.42 ± 1.46	1.96	.053
Blood group				
A	20	17		
B	21	25	2.72	.437
AB	9	15		
O	3	7		
Presence or absence of associated fibroids [n (%)]				
No	36 (67.92%)	40 (62.50%)	0.38	.540
Yes	17 (32.08%)	24 (237.50%)		
Presence or absence of endometrial polyps [n (%)]			0.12	.735
No	40 (75.47%)	50 (78.13%)		
Yes	13 (24.53%)	14 (21.87%)		
Presence or absence of adenomyosis [n (%)]				.919
No	41 (77.36%)	49 (76.56%)	0.01	
Yes	12 (22.64%)	15 (23.44%)		

E2, TG, CA125 and mass size of stage IV EMS patients were all larger than the stage III.

AMH = Anti-Mullerian Hormone, BMI = Body Mass Index, CA125 = Carbohydrate antigen 125, E2 = Estradiol, EMS = Endometriosis, HDL = High-Density Lipoprotein, LDL = Low-Density Lipoprotein, TC = Total Cholesterol, TG = Triglyceride.

### 3.3. The predictive ability of TG, E2, and CA125 for endo - and heterotaxy staging

**Figure 2.**
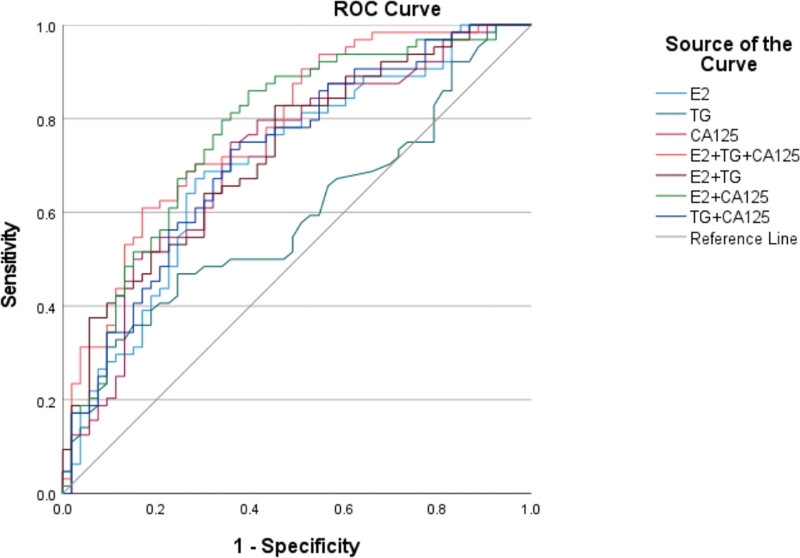
ROC curve of TG, E2 and CA125 to predict endometrial stage. When TG, E2 and CA125 are combined for staging diagnosis of EMs, the accuracy is the highest. AUC TG + E2 + CA125 = 0.777. AUC = area under the curve, E2 = estradiol, CA125 = carbohydrate antigen 125, EMS = endometriosis, ROC = receiver operating characteristic, TG = triglyceride.

The predictive ability of TG, E2, and CA125 for EMS was shown by ROC curves, which showed that the individual indexes had some value for disease prediction, and the 3 indexes could improve the staging diagnosis of EMS when combined. (AUC TG = 0.590, AUC E2 = 0.704, AUC CA125 = 0.716, AUG TG + CA125 = 0.721, AUG E2 + TG = 0.728, AUG E2 + CA125 = 0.772, AUC TG + E2 + CA125 = 0.777). Figure 2

## 4. Discussion

### 4.1. Relationship between estrogen, CA125, and endometriosis

The pathogenesis of EMS is still unclear until now, and the theory of meridional counter-current planting proposed by Sampson is the dominant theory at present, others include estrogen, oxidative stress, inflammation, and immune and genetic factors, among others.^[4,5]^ EMS is a hormone-dependent disorder in which adipose tissue can promote the interconversion between females and androgens and is an important source of estrogen in addition to the ovary.^[6]^ Maria study found that aromatase and estrogen receptor, which can promote androgen to estrogen transition, were more present in ectopic endometrium,^[7]^ and estrogen receptor was abnormally expressed in endometriosis lesions, about 140 times than in normal endometrium.^[8]^ The interaction between estrogen and its receptor enables the proliferation of endometrial cells and the reconstitution of functional endometrial vasculature, which promotes the initiation and progression of EMS. CA125 is a glycoprotein biomarker with generally low CA125 levels in the healthy population and is significantly elevated in epithelial ovarian malignancies, severe EMS, pelvic inflammation, and ruptured endometriotic cysts in response to estrogen receptors in the cytoplasm β (ER β) K can activate pro-inflammatory signals and modulate apoptosis, inflammation and aberrant neural self-pairing have been implicated in pain generation and chronic inflammation.^[9]^ E2 and CA125 of EMS patients in this study were higher than those in the non-EMS group, but there was no correlation between CA125 and E2, suggesting that CA125 was mainly related to the degree of pain and inflammation caused by EMS, and E2 was related to the occurrence and development of EMS.

### 4.2. Effects of blood lipids on EMS

The main components in blood lipids are TG and TC, which play an important role in maintaining the normal physiological activity of the body, and abnormal lipid levels are considered to be possibly associated with the existence of body oxidative stress, estrogen, inflammatory pathways, and so on, thereby affecting the occurrence and development of EMS.^[10]^ Studies have shown that patients with EMS experience changes in lipid levels during treatment with hormonal agents (e.g., oral hormonal contraceptives, gonadotropin-releasing hormone analogs, cyproterone acetate, etc).^[11,12]^

Scholars have studied the relationship between the changes in blood lipids and the occurrence, development, and degree of illness of EMS, but the results of the studies are quite different. Cell experiments, statins used for hyperlipidemia treatment can inhibit endometriotic cell growth and invasion,^[13]^ and as Melo found by examining and comparing the serum levels of HDL, LDL, TC, and TG in EMS patients with normal subjects that all lipid levels in EMS patients were significantly higher compared with normal subjects, arguing that patients with endometriosis have a poor lipid metabolism cycle.^[4]^ Melo with Verit also considered higher levels of TC and LDL in EMS patients than in non-EMS Groups.^[4,14]^ Mu study suggested that endometriosis was closely and mutually promoted with hypercholesterolemia, and that hypercholesterolemia was positively associated with the occurrence of endometriosis.^[15]^ There are also some studies holding the opposite conclusion, Gibran study showed lower LDL levels in EMS patients than controls,^[16]^ Rossi found lower BMI and less fat content in women with EMS compared to non-EMS women,^[17]^ Ozkan study found ectopic endometrial tissue can highly express Apelin, Apelin can significantly reduce BMI in EMS patients by reversing the effect of insulin resistance and promoting glucose metabolism.^[18]^ The present study showed that the occurrence of EMS had no obvious correlation with blood lipid levels and BMI, but the degree of EMS lesions was more severe in patients with high TG levels, suggesting that the TG level may be positively correlated with the degree of EMS lesions.

### 4.3. Relationship between estrogen and blood lipids

Estrogen in lipid metabolism can increase the clearance rate of chylous particle remnant particles in the liver, reduce the accumulation of lipids in the liver, promote the body’s lipid clearance, and make serum total cholesterol and low-density lipoprotein cholesterol levels decline.^[19]^ Estradiol can pass through the ER β And GPR30 activates the PI3K/ Akt signaling pathway to regulate intestinal function, reduce cholesterol accumulation, and alleviate dyslipidemia after menopause.^[20]^ Estrogen can also increase high-density lipoprotein by affecting the water salt metabolic pathway, reducing glucose tolerance, and decreasing low-density lipoprotein and cholesterol.^[21,22]^ More clinical studies have also shown that premenopausal women are less likely to develop CVD compared with men of the same age,^[23]^ and postmenopausal women have a significantly increased risk of CVD.^[24]^ Although significantly higher than non-EMS patients, blood lipids did not appear to be significantly different in the EMS patients in this study, presumably because EMS patients are generally younger at onset and lipid metabolism is not deranged with age.^[25]^

## 5. Limitations of this study

Only patients with stage III or IV EMS were included in this study, and patients with early-stage EMS were not included, which may lead to heterogeneous differences in findings. In this study, only the Chinese population was used as the research object, and the differences in ethnic origin and dietary habits were inevitable. This study is only a single center, nonlarge sample retrospective clinical study, and further multicenter, high-quality, large sample prospective clinical studies are still needed.

## Acknowledgments

We are grateful to all data collectors, Dr Yang Wang help with the preparation of figures in this paper.

## Author contributions

**Conceptualization:** Yan Lei.

**Data curation:** Rong Zheng.

**Formal analysis:** Yan Lei.

**Methodology:** Rong Zheng.

**Project administration:** Xin Du.

**Software:** Rong Zheng.

**Supervision:** Xin Du, Yan Lei.

**Visualization:** Xin Du.

**Writing – original draft:** Yan Lei.

**Writing – review & editing:** Rong Zheng.
